# Therapeutic effects of a novel BAFF blocker on arthritis

**DOI:** 10.1038/s41392-019-0051-z

**Published:** 2019-06-14

**Authors:** Bailing Zhou, Hailong Zhang, Xiaoqing Su, Yi Luo, Xiaopeng Li, Chaoheng Yu, Qibing Xie, Xuyang Xia, Gu He, Li Yang

**Affiliations:** 1 Department of Biotherapy, State Key Laboratory of Biotherapy and Cancer Center, West China Hospital, Sichuan University and Collaborative Innovation Center, Chengdu, Sichuan China; 2 Joint National Laboratory for Antibody Drug Engineering, Henan University, Kaifeng, Henan China; 3 Henan Engineering Laboratory of Antibody Medicine, Henan International United Laboratory of Antibody Medicine, Key laboratory of Cellular and Molecular Immunology, College of Medicine, Henan University, Kaifeng, Henan China

**Keywords:** Inflammation, Rheumatic diseases

## Abstract

B-cell targeted therapy is effective for autoimmune diseases such as systemic lupus erythematosus and rheumatoid arthritis (RA), although there are setbacks in RA clinical trials. In this study, we designed a novel B-cell activating factor (BAFF) antagonist: BAFF-Trap, a recombinant glycoprotein with BAFF-binding domains of two BAFF receptors (TACI and Br3) linked to Fc domain of human IgG1. Unlike TACI-Fc, BAFF-Trap bound BAFF but not APRIL (a proliferation-inducing ligand), and significantly suppressed the development of collagen-induced arthritis and adjuvant-induced arthritis. Furthermore, BAFF-Trap inhibited proinflammatory cytokine expression, ameliorated joint damage and suppressed B- and T-cell activation. BAFF-Trap reduced dendritic cells in joints, and increased regulatory T cell, regulatory B-cell, and M2 macrophage. The function of BAFF-Trap was related to inhibition of canonical and noncanonical NF-κB activation. Thus, BAFF-Trap may be a valuable agent for the effective treatment of RA.

## Introduction

Rheumatoid arthritis (RA) is a systemic autoimmune disease, which is not only related to activated T cells and macrophages, but also involved in activated B cells.^1,2^ Moreover, B-cell-depleting therapy can improve the symptoms of RA patients.^3,4^

B-cell activating factor (BAFF) is a member of the TNF superfamily expressed by macrophages, monocytes, dendritic cells (DCs), stimulated neutrophils, and stromal cells.^5^ Through binding to its receptors, BAFF regulates peripheral B-cell homeostasis and promotes B-cell survival and differentiation.^6,7^ In the peripheral lymphatic system of BAFF^−/−^ mice, mature B lymphocytes are significantly decreased.^8^ Conversely, overproduction of BAFF in transgenic (TG) mice leads to an enlarged spleen, increased number of mature B cells, and generation of autoimmune diseases resembling RA and systemic lupus erythematosus (SLE).^9^ In addition, BAFF can regulate the function of T cells by enhancing the production of co-stimulatory signals to T cells in conjunction with T cell receptor.^10,11^ Thus, BAFF may be an ideal therapeutic target for autoimmune diseases, such as RA.

Several strategies have been used to block BAFF activity, including blocking antibodies,^4,12,13^ decoy-soluble receptors,^14,15^ and gene silencing.^11^ Several BAFF-targeted drugs have been developed for phase II/III clinical research, such as belimumab, tabalumab, and atacicept.^16–18^ Belimumab is the first B-cell-targeted drug that is beneficial in treating SLE, which has been approved by the US Food and Drug Administration (FDA).^19^ Latest research data showed belimumab can significantly improve disease activity in SLE patients from North East Asia.^16^ Although belimumab can significantly deplete naive B cells, activated B cells, and plasma cells, it has suffered some setbacks in clinical phase III studies of RA, which may relate to patient heterogeneity.^19,20^ In addition, previous animal studies have demonstrated that administration of decoy BAFF receptors (TACI-constant region (Fc) and Br3-Fc) effectively inhibits the BAFF-signaling pathway, and then suppresses B-cell maturation and survival, as well as reducing circulating immunoglobulins.^14,15^ However, both antagonists do not reduce the number of memory B cells that are important for long-term humoral immunity.^21^ Moreover, TACI-Fc can also bind to a proliferation-inducing ligand (APRIL), although the functions of APRIL are poorly understood.^22^ Recent studies demonstrate that administration of TACI-Fc induces side effects in RA patients and clinical trials of TACI-Fc in RA patients show no difference when compared with control groups, which may be related to the combination between TACI and APRIL and patient heterogeneity.^23,24^ Because the binding affinity between Br3 and BAFF is relatively weak, the effectiveness of Br3-Fc has been limited in preclinical and clinical research.^13^ Taken together, these reports underline the need to optimize the efficiency of blocking molecules. Here, we engineer a high-affinity BAFF antagonist (BAFF-Trap) that has improved the deficiencies of TACI-Fc and Br3-Fc. Moreover, BAFF-Trap shows no side effects and effectively inhibits arthritis development and joint destruction in collagen-induced arthritis (CIA) mice and adjuvant-induced arthritis (AIA) rats, which are associated with regulating disease-related immune cells and inhibiting the generation of proinflammatory cytokine.

## Materials and methods

### Mice

Male 8-week-old DBA/1j mice were purchased from Shanghai SLAC Laboratory Animal Co. Ltd (Shanghai, China). Male 8-week-old Lewis rats were purchased from Beijing Vital River Laboratories Animal Technology Co. Ltd (Beijing, China). All mice were maintained in a pathogen-free animal facility. All experimental procedures and animal care were performed in accordance with the regulations of the Animal Care Committee of Sichuan University.

### Cell lines

The Chinese hamster ovary cells (CHO cells) and Burlitt lymphoma and lymphoblastoid cell line (Raji) were obtained from ATCC (Virginia, USA). CHO cells were maintained in F-12 medium (Gibco, California, USA). However, when CHO cells transfected with recombinant plasmids were used to produce targeted protein, the F-12 medium was supplemented with 10% fetal bovine serum (FBS) (Gibco, California, USA). Raji cells were maintained in Rosewell Park Memorial Institute-1640 medium supplemented with 10% FBS.

### Expression and purification of BAFF-Trap

First, CHO cells were transfected with the recombinant plasmid, pEF1/V5-BAFF-Trap. Second, cloned strains that stably and efficiently expressed BAFF-Trap were obtained through G418 (2 μg/ml) (Sigma-aldrich, Saint Louis, USA) selection. After intermediate culture of BAFF-Trap-expressing cells, the culture supernatant was collected and loaded onto a protein-A affinity column (GE, Pittsburgh, USA) equilibrated with binding buffer (20 mm phosphate buffer, pH 7.2). The column was washed with binding buffer and further washed with 0.1 m citrate sodium buffer (pH 3.6). Then the pH of the solution harboring BAFF-Trap was adjusted to 7.2 with Tris buffer. After dialysis with 20 mm PB (pH 7.2), the protein solution was loaded onto a SP chelating Sepharose column (GE, Pittsburgh, USA) equilibrated with 20 mm PB (pH 7.2). The column was washed with 20 mm (pH 7.2) and the hybrid protein was detached with 20 mm PB (pH 7.2) and 100 mm NaCl buffer. The target protein was eluted with 20 mm PB (pH 7.2) and 300 mm NaCl buffer. Eventually, the protein solution was dialyzed with phosphate-buffered saline (PBS).

### Western blot analysis

The characterization of BAFF-Trap was confirmed by western blot analysis. First, purified BAFF-Trap was electrophoresed by 12% sodium dodecyl sulfate polyacrylamide gel electrophoresis (SDS-PAGE) and transferred onto a polyvinylidene difluoride membrane (Bio-Rad, Richmond, USA). After being washed with PSB tween-20 (PBST), the membrane was blocked with 5% skimmed milk powder for 2 h at 37 °C. Then the blots were incubated with goat anti-Br3 antibody or goat anti-TACI antibody (1:1000) (R&D Systems, Minnesota, USA) at 4 °C overnight. The secondary antibody was rabbit anti-goat IgG antibody (horseradish-peroxidase (HR)–conjugated, 1:5000) (ZSGB-BIO, Beijing, China). After each incubation, the membrane was washed five times with PBST (5 min per time). Finally, the protein bands were visualized through a chemiluminescence detection system (Pierce, Rockford, USA).

### Enzyme-linked immunosorbent assay (ELISA)

The binding abilities and affinities of BAFF-Trap to BAFF were measured by a specific and sensitive ELISA (R&D Systems, Minnesota, USA), which detects unbound human BAFF at 25 pM (R&D Systems, Minnesota, USA) mixed with BAFF-Trap (ranging in concentration from 0.04 nm to 655.36 nm) incubated overnight at room temperature. The same serial amounts of TACI-Fc and Br3-Fc (R&D Systems, Minnesota, USA) were added as controls.

### In vitro pull-down assay

Recombinant protein (200 ng BAFF or APRIL) in 200 μl PBS was incubated with 1 μg of BAFF-Trap, TACI-Fc, or Br3-Fc at 4 °C overnight. Then, 50 μl of protein-A agarose beads was added to the mixture and incubated at 4 °C for 2 h. After centrifugation at 20,000 × *g* for 3 min, the precipitation containing protein-A conjugated samples was obtained and washed with PBS. The precipitation was eluted with PBS and mixed with SDS-PAGE loading buffer, which was then separated by SDS-PAGE and detected with western blot analysis. The first antibodies were goat anti-human TACI antibody (R&D Systems, Minnesota, USA), rat anti-mouse/human BAFF antibody and mouse anti-human APRIL antibody (R&D Systems, Minnesota, USA), respectively. The corresponding secondary antibodies were HRP-conjugated rabbit (anti-goat, rat and mouse) IgG antibody (ZSGB-BIO, Beijing, China).

### Raji cell-binding assay

The expression of TACI and Br3 in Raji cells were validated by flow cytometry. Goat anti-TACI or goat anti-Br3 (10 μg/ml) was added onto Raji cells (1 × 10^6^) and then incubated for 60 min at room temperature. Raji cells were washed with PBS and then incubated with phycoerythrin (PE)-conjugated rabbit anti-goat IgG (BD Biosciences, USA) antibody for 45 min. BAFF-Trap blocking of BAFF binding to Raji cells was also assessed by flow cytometry. In all, 10 μg/ml human BAFF was incubated with various concentrations of BAFF antagonists (5, 10, 20, or 50 μg/ml) for 60 min at 4 °C. The volume was 0.1 ml. Then the mixture was added to Raji cells (2 × 10^5^) for 30 min at 4 °C. Raji cells were washed with PBS and then incubated with PE-conjugated anti-BAFF antibody (BD Biosciences, New Jersey, USA) for 45 min at 4 °C in the dark. FACSCalibur™ flow cytometer (BD Biosciences, New Jersey, USA) was used in flow cytometry to detect the samples.

### BAFF-induced proliferation assay

Raji cell proliferation was measured by CCK-8 cell counting kit (Dojindo, Kumamoto, Japan). A total of 5 × 10^3^ Raji cells were plated per well of a 96-well flat bottom plate and incubated for 72 h with vehicle or mixtures of BAFF antagonists (the concentrations ranging from 0.68 nm to11 mm) with 1 μg/ml of BAFF and/or 10 μg/ml of anti-human IgM (R&D Systems, Minnesota, USA), followed by addition of 10 μl of CCK-8 buffer for 2 h. The absorbance of the solution at 450/570 nm was measured by UV spectrophotometer (Shimadzu, Kyoto, Japan).

### Generation of CIA model and treatment regimes

To generate the CIA model, bovine CП (Chondrex, WA, USA) was dissolved at 2 mg/ml in 0.05 m acetic acid by stirring overnight at 4 °C and mixed with an equal volume of complete Freund’s adjuvant (Chondrex, WA, USA). When the mixture was emulsified thoroughly, male DBA/1j mice were intradermally injected with 100 μl of this emulsion on day 0 at the base of the tail. On day 21, mice were given a second immunization intradermally with 100 μg of bovine CII emulsified in incomplete Freund’s adjuvant (Chondrex, WA, USA). After the booster immunization, the severity of CIA was evaluated three times a week for joint edema, erythema, and flexion. Each of the paws was scored from 0 to 4 as previously described.^25^ All four paws were scored, and the maximal clinical score per mouse was 16. The body weights of mice were also measured on the same days.

On day 31, most mice showed features of CIA, and were randomly divided into five groups as follows: treated with (i) PBS, (ii) 6 mg/kg BAFF-Trap, (iii) 30 mg/kg BAFF-Trap, (iv) control hIgG (10 mg/kg), or (v) Entanercept (6 mg/kg). All drugs were injected intraperitoneally three times per week, for a total of 18 times. On day 77, paw swelling was measured with a slide gauge, and its degree was assessed by the increase in thickness compared with the thickness of the paws of normal mice.

### Generation of AIA model and treatment regimes

Arthritis was induced by Freund’s complete adjuvant (CFA) inoculation of the rats. Rats were injected intradermally at the base of the tail with 0.1 ml CFA (Chondrex, WA, USA).

On day 17, most rats showed features of AIA, and were randomly divided into three groups as follows: treated with (i) PBS, (ii) 4.5 mg/kg hIgG, (iii) 1.5 mg/kg BAFF-Trap. All drugs were injected subcutaneously, once every 2 days, for a total of 15 times. On day 50, paw swelling was measured with a slide gauge, and its degree was assessed by the increase in thickness compared with the thickness of the paws of normal mice.

### Histological assessment

On day 77, mice were killed and joint tissues were harvested. After fixation with 10% formalin/PBS for 24 h, the tissues were decalcified with ethylenediaminetetraacetic acid for 21 days and embedded in paraffin. Then, tissue sections were obtained at 4-μm thickness and stained with hematoxylin-eosin staining (HE). Histological scores of joint pathology were measured by two independent observers as previously described.^25^

### Nuclear magnetic resonance imaging (MRI) assessment

Rats were imaged with a Bruker BioSpec 7.0 T MR microimaging system (Bruker, Karlsruhe, Germany) in West China Hospital, Sichuan University (Chengdu, China), using a Bruker rat brain coil array located over both knees. Rats were anesthetized with 1.5–2% isoflurane by nosecone and were physiologically monitored. After the acquisition of a scout view, sagittal, axial and coronal images of both knees were obtained from each animal with a field of view of 40 mm, a 256 × 256 pixel matrix, and a slice thickness of 1.0 mm using a TurboRARE-T2 sequence with TR 2500 msec, TE 33 msec.

### Measurement of BAFF, anti-CII antibody, and inflammatory-related cytokines levels in serum

The levels of TNF-α, IL-1β, IL-6, and BAFF in serum were measured with ELISA kits (R&D Systems Minnesota, USA) according to the manufacturer’s instructions.

In addition, serum levels of anti-CII antibodies were detected using an established ELISA. In brief, the wells of a 96-well plate (Dynatech Laboratories, Chantilly, USA) were coated with 500 ng of purified CII collagen (Sigma-Aldrich, Saint Louis, USA) in 100 μl PBS and incubated at 4 °C overnight. After being washed with PBST three times, the plate was blocked with 5% skimmed milk powder in PBST. The serum samples were obtained at day 42 after treatment and diluted to 1:10,000 and 1:100,000. The diluted serum samples were added to the plate, which was then incubated at 37 °C for 1 h. After washing the plate three times with PBST, total anti-CII antibody levels were detected with HRP-conjugated goat anti-mouse IgG (Southern Biotech, Birmingham, AL), and the IgG subclasses levels were determined using HRP-conjugated goat anti-mouse IgG1, IgG2a, or IgG2b (Southern Biotech, Birmingham, AL). Then, the substrate was developed with 50 μl of 3,3′,5,5′-tetramethylbenzidine (Southern Biotech, Birmingham, AL, USA) for 10 min, and the reaction was stopped with 50 μl of 0.5 m H_2_SO_4_. The absorbance was measured at 450 nm and 570 nm with an ELISA reader (Thermo Scientific, Multiskan Mk3, USA).

### Reverse transcription and semiquantitative PCR

Total RNA samples from joint tissues were obtained using TRIzol (Invitrogen, California, USA), according to the manufacturer’s instructions. Complementary DNA and PCR were performed with PrimeScript One step RT-PCR kit (Takara, Dalian, China), according to the manufacturer’s instructions. The reactions were validated under the following conditions: first 50 °C for 30 min, then denaturation at 94 °C for 2 min, followed by 98 °C for 10 s, with a final step at 68 °C for 30 s. The amplifications were conducted for 25–30 cycles for the analyzed cytokines. The products were detected by agarose gel electrophoresis using Molecular Imager Gel DOCTM XR + system (Bio-Rad, Richmond, USA), and the band intensity was quantified by Image Lab™ software (Bio-Rad, Richmond, USA). Primer sequences were listed in Supplemental Table I. As an internal control, β-actin primers were used to normalize sample amounts.

### Analysis of immune cells from spleen, axillary and peripheral lymph nodes, and joints by flow cytometry

On day 77, mice were killed and spleens, axillary and peripheral lymph nodes, and joints were obtained. Subsets of B lymphocytes from spleen were analyzed by fluorescence activated cell sorting (FACS) (BD Biosciences, New Jersey, USA) after incubation with the following antibody combinations: IgM-FITC/CD19-PE, IgD-FITC/CD19-PE, CD23-FITC/CD19-PE, CD45R-FITC/CD19-PE, and CD27-FITC/CD19-PE. Subsets of T lymphocytes from lymph nodes were stained with the following antibody combinations: CD3-PE/CD4-perCP, CD62L-PE/CD4-perCP, and CD69-PE/CD4-perCP, and then detected by FACS. DCs from joints were analyzed after staining with CD11c-PE/CD11b-FITC antibody combinations, respectively. All antibodies were anti-mouse and purchased from BD Biosciences (New Jersey, USA). Analysis of regulatory T (Treg) cells (CD4^+^CD25^+^Foxp3^+^) from joints was performed using FACS after being stained with the Treg detection kit (Miltenyi Biotec Technology & Trading, Belgish-Gladbach, Germany) according to the manufacturer’s instructions.

### Intracellular cytokine staining

Immune cells were obtained from joints as mentioned above. Then, they were incubated with PE-conjugated anti-mouse CD4 antibody for 30 min at 4 °C to stain the cell surface. After being washed with PBS, cells were fixed with 2% paraformaldehyde, permeabilized with 1% Triton-X100, and stained with anti-IFN-γ-FITC or anti-IL-17A-FITC to detect Th1 cells and Th17 cells by FACS. Regulatory B (Breg) cells from joints were also detected with PE-conjugated anti-mouse CD19 and FITC-conjugated IL-10 as mentioned above. The corresponding isotype controls were purchased from BD Biosciences (New Jersey, USA).

### Analysis of canonical and noncanonical NF-κB-signaling pathways by western blot

In the presence of hBAFF (100 ng/ml) and anti-hIgM (10 μg/ml), Raji cells expressing BAFF receptors were cultured for 48 h with BAFF-Trap at various concentrations (1.56, 6.25, 25, and 100 μg/ml). Then, cells were homogenized in radioimmunoprecipitation assay lysis buffer and centrifuged for 10 min at 17,000 × *g* to obtain cell lysate supernatants. The Bradford method was used to determine protein concentrations of the supernatants. The same concentration of protein samples was used to analyze the canonical and noncanonical NF-κB-signaling pathways by western blotting using the above method. The following antibodies purchased from Cell Signaling Technology (Boston, USA) were used as primary antibodies: anti-human phosphorylated Akt, anti-human phosphorylated NIK, anti-human IKKα, anti-human phosphorylated IKKα, anti-human phosphorylated Ikβα, anti-human P100, anti-human phosphorylated P100, anti-human P52, anti-human Bcl-2, and anti-human GAPDH. HRP-conjugated rabbit anti-goat IgG antibody (ZSGB-BIO, Beijng, China) was used as a secondary antibody. The signal was visualized using an enhanced chemiluminescence scanner (Pierce, Rockford, USA).

### Statistical analysis

SPSS 17.0 was used for statistical analysis. Data were presented as means ± S.D. or medians. Differences between groups were determined by performing analysis of unpaired Student’s *t* test or one-way ANOVA. The difference was regarded statistically significant if *P* < 0.05.

## Results

### Generation and characterization of BAFF-Trap

**Fig. 1:**
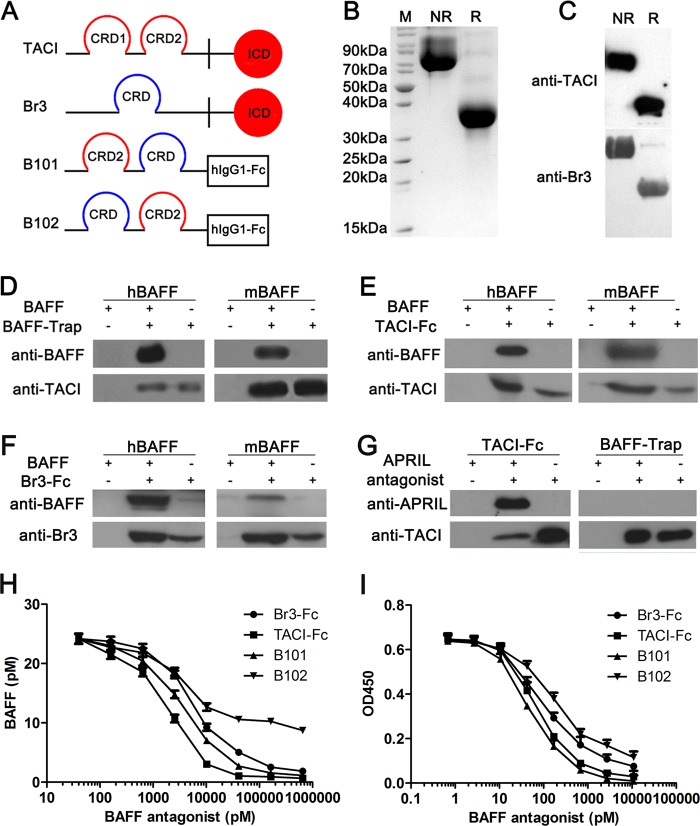
Generation and Biochemical characteristics of BAFF-Trap. **a** Schematic diagrams of TACI, Br3 and two BAFF-Trap variants (B101 and B102). B101 was designed by orderly connecting CRD2 of TACI, CRD of Br3, and Fc domain of hIgG1, whereas the sequence of CRD2 of TACI and CRD of Br3 was reversed in B102. **b** SDS-PAGE analysis of purified BAFF-Trap protein (B101). After purified with gel column, 10 μg of BAFF-Trap was verified at reduced condition and unreduced condition by SDS-PAGE, respectively. M means protein markers. R represents reduced buffer of BAFF-Trap solution, which was used to detect the BAFF-Trap monomer (36.6KD). Although NR means unreduced buffer, which meant the BAFF-Trap dimer (72.8 KD). **c** Western blot analysis of purified BAFF-Trap protein (B101) demonstrating BAFF-Trap specifically binds to anti-human TACI antibody (top) and anti-human Br3 antibody (bottom). Binding assay between BAFF-Trap (B101) **d**, TACI-Fc **e**, or Br3-Fc **f** and BAFF (hBAFF and mBAFF) through Immunoprecipitation (IP). After incubated with hBAFF or mBAFF overnight, BAFF antagonists binding to BAFF were collected by protein-A agarose beads, and then BAFF was verified by WB through anti-BAFF antibody. **g** Binding assay between TACI-Fc, or BAFF-Trap and APRIL through IP. **h** The binding affinity of BAFF antagonists to BAFF. Several concentrations of BAFF antagonists were mixed with hBAFF overnight, then unbound BAFF was detected by ELISA. **i** BAFF antagonists inhibit Raji cell proliferation induced by BAFF and anti-IgM. Several concentrations of BAFF antagonists along with BAFF and anti-IgM were treated to Raji cells for 72 h, and then the cell number was detected by CCK-8

To design a double decoy receptor, BAFF-Trap, containing sequences of TACI and Br3, the cysteine-rich domain (CRD) 2 of TACI and the CRD of Br3 was fused to the Fc of human IgG1, which was then cloned into a pEF1/V5 plasmid (Fig. 1a). Moreover, two BAFF-Trap variants were designed, termed B101 and B102, and then purified by Protein-A affinity chromatography and ion exchange chromatography (SP Sepharose), with a purity almost 95% (Fig. 1b). Western blot analysis showed BAFF-Trap could specifically bind to anti-human TACI antibody and anti-human Br3 antibody (Fig. 1c).

In vitro pull-down assays revealed BAFF-Trap could bind to mouse and human BAFF (mBAFF and hBAFF, Fig. 1d). Consistent with previous studies,^26,27^ TACI-Fc and Br3-Fc could bind to BAFF (Fig. 1e, f). Furthermore, TACI-Fc could also bind to APRIL, which is dependent on the CRD2 of TACI, as confirmed by in vitro pull-down assays (Fig. 1g). Although BAFF-Trap contains the CRD2 of TACI, our findings showed that BAFF-Trap could not bind to APRIL (Fig. 1g).

Equilibrium binding assays were conducted to determine the binding affinity of BAFF-Trap for BAFF. The binding BAFF affinities of TACI-Fc and Br3-Fc were 1.94 nm and 5.87 nm, respectively, yet B101 had a *K*_D_ of 3.62 nm, which was higher than for B102 (Fig. 1h). Thus, B101 was termed BAFF-Trap and used for all further experiments.

To determine whether BAFF-Trap could efficiently interrupt BAFF binding and activation of its receptors, BAFF-Trap was added to BAFF-cultured Raji cells that expressed TACI and Br3 (Supplemental Fig. 1A, B). Although B101 had an appreciably higher *K*_D_ than TACI-Fc, it had a significantly greater blocking ability for BAFF binding to Raji cells, which was dose-dependent (Supplemental Fig. 1C). The ability of BAFF-Trap-blocking BAFF binding to Raji cells was higher than that for Br3-Fc, and B102-blocking ability was the lowest among the BAFF antagonists tested (Supplemental Fig. 1C). Accordingly, B101 almost completely blocked activated Raji cell proliferation, with an IC_50_ of 50.62 nm (Fig. 1i). Compared with B101, both TACI-Fc and Br3-Fc also potently inhibited BAFF and anti-IgM induced proliferation in Raji cells, with a respective IC_50_ of 69.55 nm and 93.42 nm, which were lower than for B101, yet higher than for B102 (165.4 nm) (Fig. 1i).

### BAFF-Trap dramatically inhibits disease development in CIA and AIA model

**Fig. 2:**
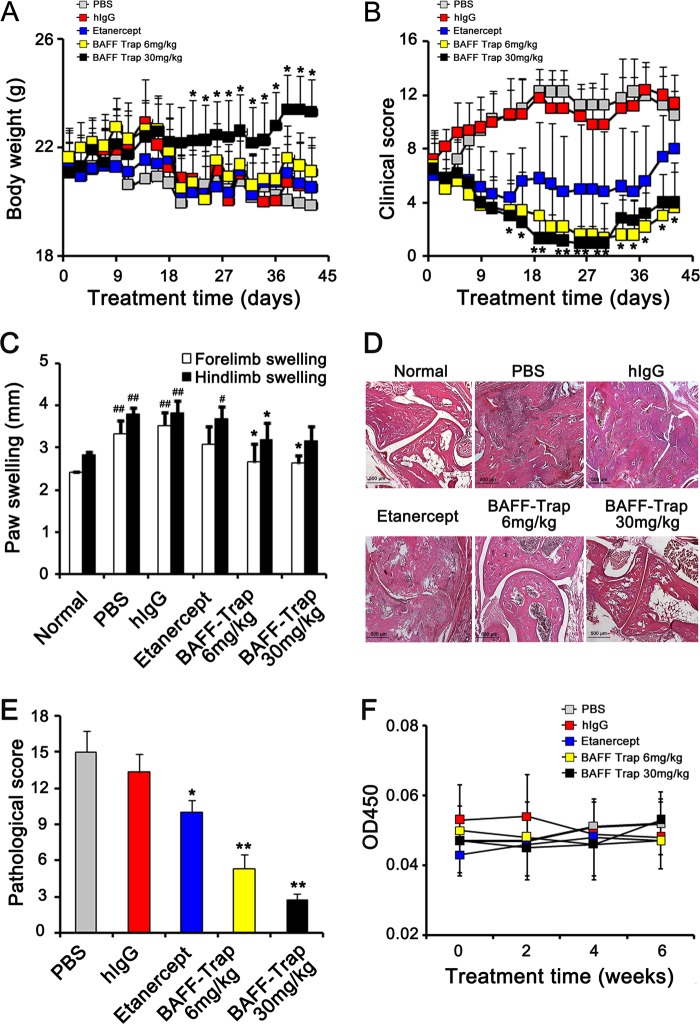
BAFF-Trap inhibits disease development and ameliorates pathological damage in CIA mice. **a** Body weight of CIA mice after treatment with PBS, hIgG, Etanercept, or BAFF-Trap (6 mg/kg and 30 mg/kg). Bars represent mean ± S.D. (*n* = 5). **b** Clinical scores of CIA mice. Bars represent mean ± S.D. (*n* = 5). **c** Paw swelling of with indicated treatments. On day 42 after treatment, paw swelling of CIA mice was measured with a slide gauge. Bars represent mean ± S.D. (*n* = 5). **d** HE analysis of representative joint sections from experiments in **a**. At day 42 after treatment, the joints were separated to obtain ankle sections, which were then stained with HE. Magnification = × 40, scale bar is 500 μm. **e** Pathological scores of joint sections in **d**. Bars represent mean ± S.D. (*n* = 6). **f** The titer of anti-BAFF-Trap antibody in serum. The serum was collected every other week after treatment, and then the titer of anti-BAFF-Trap antibody was detected by ELISA. *P < 0.05 versus PBS; **P < 0.01 versus PBS; ^#^*P* < 0.05 versus normal; ^##^*P* < 0.01 versus normal

To explore the value of BAFF-Trap as a BAFF antagonist, we evaluated its ability to inhibit disease development in CIA model. Compared with hIgG and Etanercept treatment, BAFF-Trap significantly inhibited the development of CIA and alleviated the clinical score. However, there were no obvious differences between the 6 and 30 mg/kg of BAFF-Trap treated groups (Fig. 2a, b, Supplemental Fig. 2A). To determine the minimal effective dosage, we examined the ability of 0.67, 2, and 6 mg/kg of BAFF-Trap in CIA. The results showed that 2 mg/kg of BAFF-Trap still effectively inhibited the development of CIA, whereas 0.67 mg/kg of BAFF-Trap did not (Supplemental Fig. 2B–D). Moreover, the ability of B101 was higher than that of B102 (Supplemental Fig. 2B–D). On day 41 after treatment, paw swelling of mice was measured by slide gauge. In comparison with PBS and hIgG treatment, paw thickness was reduced by 50% in the 6 mg/kg BAFF-Trap group and 60% in the 30 mg/kg BAFF-Trap group (Fig. 2c). Importantly, there were differences between the Etanercept and BAFF-Trap treatment groups, paw swelling data showed that BAFF-Trap could significantly reduce 14% paw thickness than Etanercept (Fig. 2c). HE staining assay showed there were no obvious synovial hyperplasia, cartilage damage, lymphocyte infiltration, and bone erosion in BAFF-Trap treated groups, which was almost normal (Fig. 2d, e). These findings indicated that BAFF-Trap effectively inhibited the development of CIA and ameliorated disease severity.

Moreover, the potential toxicity of BAFF-Trap was analyzed. Drug toxicity indices including body weight, renal function, and damage to major organs were closely monitored after mice received indicated treatments. Overall, no marked differences were observed among all groups, and no pathological damages were observed in tissue sections of major organs stained with HE. Moreover, the immunogenicity of BAFF-Trap was measured using serum from mice treated with BAFF-Trap. ELISA detected no antibodies against BAFF-Trap (Fig. 2f).

**Fig. 3:**
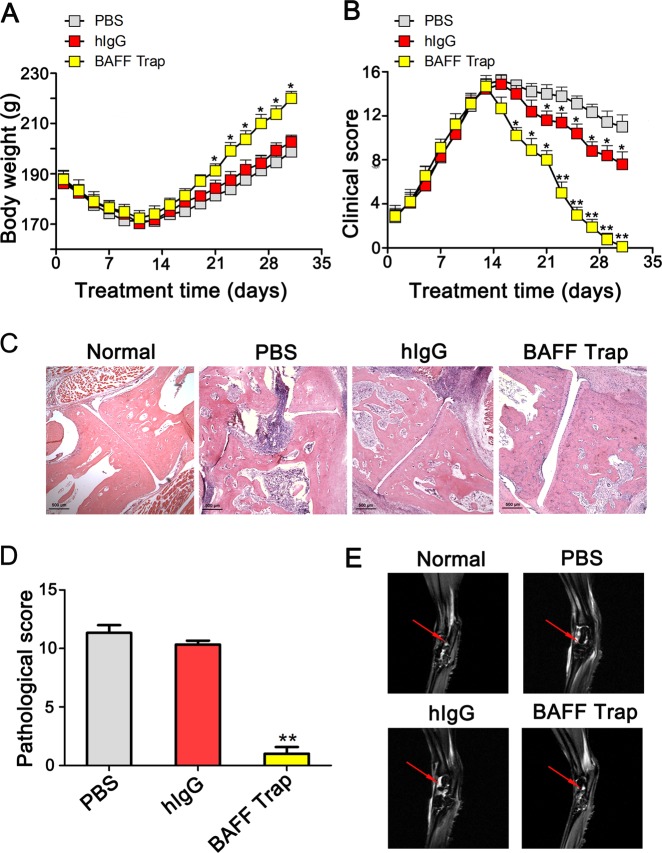
BAFF-Trap inhibits disease development and ameliorates pathological damage in AIA rats. **a** Body weight of AIA rats after treatment with PBS, hIgG, or BAFF-Trap. Bars represent mean ± S.D. (*n* = 9). **b** Clinical scores of AIA rats. Bars represent mean ± S.D. (*n* = 9). **c** HE analysis of representative joint sections. Magnification = × 40, scale bar is 500 μm. **d** Pathological scores of joint sections in **c**. Bars represent mean ± S.D. (*n* = 6). **e** Analysis of rat ankle joint by MRI. The ankle joints were assessed by MRI (T2-weighted MR-images, TR/TE = 2500 msec/33 msec). The joint effusion was indicated by a red arrow. *P < 0.05 versus PBS; **P < 0.01 versus PBS

Besides CIA model, we also evaluated the therapeutic effect of BAFF-Trap in AIA model. Similarly, BAFF-Trap significantly inhibited the development of AIA and alleviated the clinical score compared with PBS and hIgG (Fig. 3a, b), the most effective dosage is 1.5 mg/kg in experimental groups (Supplemental Fig. 3A, B). Synovial hyperplasia, cartilage damage, lymphocyte infiltration, and bone erosion of rat joints in BAFF-Trap treated groups were alleviated consistently, which were examined by HE staining (Fig. 3c, d). To investigate the effect of BAFF-Trap on the repair of rat joints, we examined the ankle joints of rats with MRI at the end of experiment. The results showed that there were obvious bone destruction, cysts and joint effusion in PBS group and hIgG group, whereas BAFF-Trap could repair damaged bone and eliminate joint effusion (Fig. 3e).

### BAFF-Trap regulates the development of B cells and the generation of autoantibody by reducing BAFF levels

**Fig. 4:**
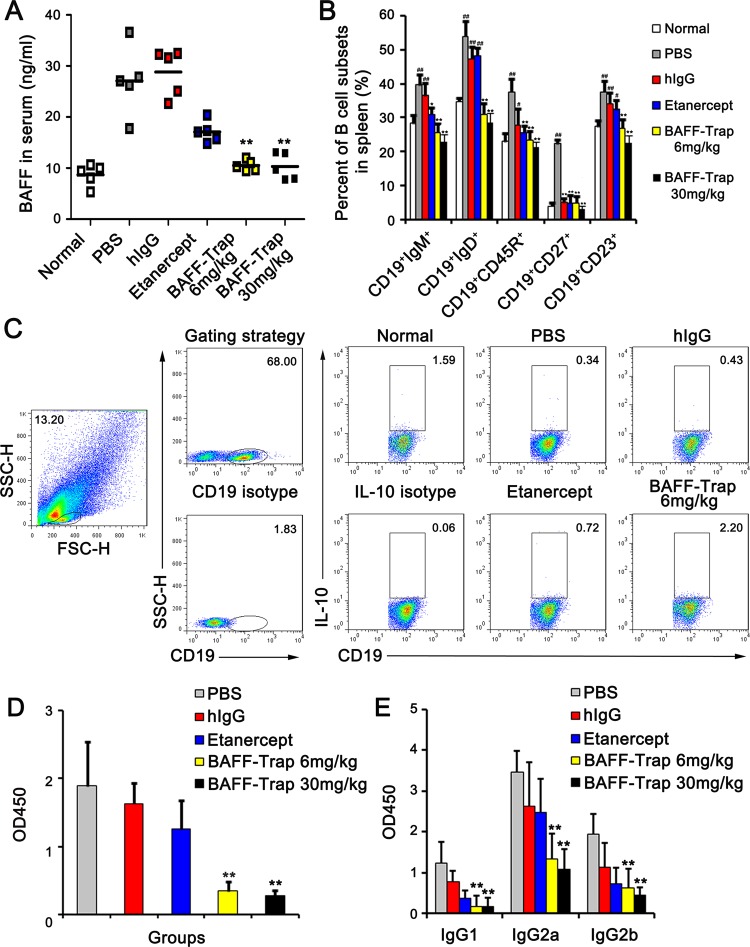
BAFF-Trap regulates B cells development and autoantibody production through reducing BAFF levels. **a** The protein levels of BAFF in serum from CIA mice treated with PBS, hIgG, Etanercept, and BAFF-Trap (6 mg/kg and 30 mg/kg). On day 42 after the indicated treatments, the serum of CIA mice was obtained. The levels of BAFF were detected by ELISA. **b** Percentage of B-cell subsets in spleen. On day 42 after the indicated treatments, mice were killed to obtain splenocytes. Then the splenocytes were incubated with corresponding antibodies to detect the subsets of B cells. Bars represent mean ± S.D. (*n* = 5). **c** Representative FACS analysis of Breg cells from joints. After digested by collagenase type IV, the cells in joint were obtained and incubated with corresponding antibodies to detect Breg cells. CD19^+^IL10^+^ cells meant Breg cells. Bars represent mean ± S.D. (*n* = 5). **d** The level of anti-CII IgG in serum obtained on day 42 after treatment. Serum was diluted to 1:10,000, and then anti-CII IgG concentrations were determined by ELISA. **e** The concentrations of anti-CII isoforms (IgG1, IgG2a, and IgG2b) on day 42 after indicated treatments. **e** TNF-α, **f** IL-1β, and **g** IL-6 concentration in serum from CIA mice, measured once every 7 days beginning on the first day of immunization. Bars represent mean ± S.D. (*n* = 5). ^#^*P* < 0.051 versus normal; ^##^*P* < 0.01 versus normal; *P < 0.05 versus PBS; **P < 0.01 versus PBS

BAFF levels are elevated in the serum of RA patients and mice with CIA.^11,19^ To investigate whether BAFF-Trap reduced the amount of BAFF in CIA mice, we measured BAFF levels in the serum of CIA mice at day 42 after treatment. Significantly decreased BAFF serum levels were observed compared with controls, which were similar to normal mouse levels (Fig. 4a). Moreover, we detected the BAFF gene in joints by semiquantitative PCR assay using β-actin as a control. In accordance with the previous results, BAFF gene expression in the PBS treatment group was sixfold higher than that of normal mice, and BAFF-Trap markedly reduced BAFF gene expression (Supplemental Fig. 4A, B). However, compared with PBS treatment, BAFF-Trap did not reduce APRIL gene expression (Supplemental Fig. 4A, B).

BAFF plays an important role in B-cell activation, survival, and development. Overexpression of BAFF in TG mice resulted in the splenic expansion of mature B-1 and B-2 B cells, which are important features in RA patients and CIA mice.^9^ To determine the effect of BAFF-Trap on B cells, we measured B-cell subtypes in the spleen of mice treated with BAFF-Trap by FACS. There was no significant difference in the weight and volume of spleen of mice among these groups. However, the number of total splenocytes in mice treated with BAFF-Trap was 2.4-fold lower than in control mice, and the number of B cells (CD19^+^CD45R^+^) was significantly reduced in the spleen (Fig. 4b). In addition, there was a significant decrease in the number of immature transitional B cells (CD19^+^IgM^+^), from 37.34 ± 3.61% (control mice) to 24.62 ± 2.89% (6 mg/kg BAFF-Trap) and 22.07 ± 1.58% (30 mg/kg BAFF-Trap) (Fig. 4b). This suggests transitional B cells in the spleen depend on BAFF for their development and survival.

In the BAFF-Trap-treated groups, there was a 1.2-fold (6 mg/kg BAFF-Trap) and 1.7-fold (30 mg/kg BAFF-Trap) reduction in splenic naive B cells (CD19^+^ IgD^+^), whereas the number of naive B cells was relatively unaffected in Etanercept-treated mice (Fig. 4b). There was also a decrease in CD19^+^ activated B-cell expression of CD23, present on the surface of follicular B cells. BAFF-Trap effectively inhibited autoantibody production. The number of plasmablasts and plasma cells (CD19^+^CD27^+^) was reduced from 16.15 ± 2.48% in the PBS-treated group to normal levels (5.26 ± 3.63%) in BAFF-Trap-treated groups (Fig. 4b). These findings suggest BAFF is important for the development of these B-cell subtypes, and that BAFF-Trap significantly suppressed the elevated numbers of autoreactive B cells in CIA mice.

Recent studies identified IL-10-secreting B cells that inhibit immune responses and autoimmune diseases such as RA.^28^ Semiquantitative reverse transcription polymerase chain reaction (RT-PCR) assay showed that IL-10 gene expression was markedly elevated in the joints of BAFF-Trap-treated mice (Supplemental Fig. 4A, B). Joints were digested with collagenase type IV to obtain leukocytes that were stained with fluorescently labeled anti-CD19 and anti-IL-10 antibodies. In the BAFF-Trap-treated group, sevenfold increased numbers of IL-10-secreting B cells were observed in the joint (Fig. 4c). Interestingly, although BAFF and IL-12 could induce B cells to produce IL-10, their expression was significantly reduced in CIA joints (Supplemental Fig. 4A, B).

The production and enrichment of anti-collagen type II (CII) autoantibodies that contribute to disease development are important features of CIA.^23^ To determine whether BAFF-Trap inhibited the level of anti-CII autoantibodies, serum was obtained from CIA mice on day 42 after treatment, and used to detect the titer of total IgG and all IgG isoforms against bovine CII. BAFF-Trap markedly decreased the serum level of anti-CII IgG, and mice treated with BAFF-Trap had reduced levels of CII-specific IgG1, IgG2a, and IgG2b antibodies than control mice (Fig. 4d, e). Moreover, IgG2a had the largest proportion among all the IgG isoforms. These findings indicated BAFF-Trap suppressed the development of CIA, in part owing to decreased BAFF levels in mice and the subsequent inhibition of anti-CII antibody production.

### BAFF-Trap regulates inflammatory cytokine production

**Fig. 5:**
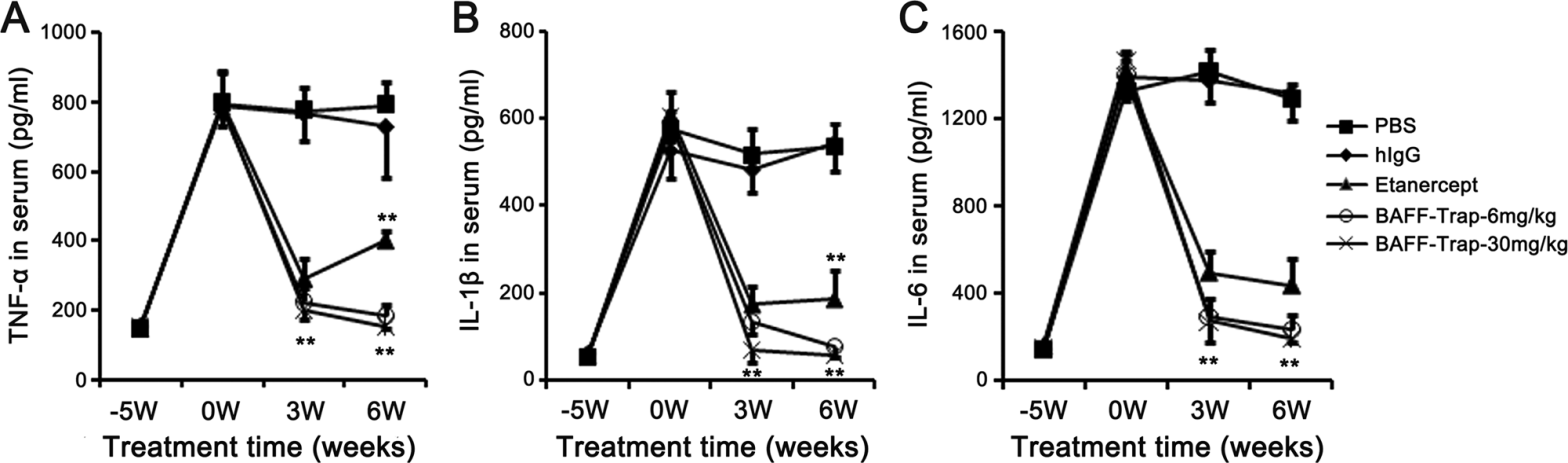
BAFF-Trap reduces the levels of proinflammatory cytokines in CIA mice. **a** TNF-α, **b** IL-1β, and **c** IL-6 concentration in serum from CIA mice. The serum was obtained from the first day of immunization, then the levels of proinflammatory cytokines were detected by ELISA. Bars represent mean ± S.D. (*n* = 5). **P < 0.01 versus PBS

B cells can regulate inflammatory cell production and migration through secreting inflammatory-related cytokines. Proinflammatory cytokines play an important role in CIA pathogenesis.^29^ To detect changes in proinflammatory cytokines, we performed a time-course study on the expression of TNF-α, interleukin (IL)-1β, and IL-6 in the serum and found that the concentrations of these cytokines were significantly elevated in PBS and hIgG treated groups, which lasted until the end of the experiment (Fig. 5a–c). However, BAFF-Trap and Etanercept treatment effectively decreased cytokine levels, and there was no difference in levels between these groups until 6 weeks after the first immunization (Fig. 5a–c). Thus, BAFF-Trap significantly suppressed the long-term production of proinflammatory cytokines that mediate CIA.

Similar to protein levels in serum, TNF-α, IL-1β, and IL-6 gene expression was markedly decreased in mice treated with BAFF-Trap compared with control mice (Supplemental Fig. 4A, B). Furthermore, mRNA levels of Th1 cytokines (IL-2, IFN-γ, and IL-12) and Th17 cykotine (IL-17) were markedly reduced in the joints of mice treated with BAFF-Trap. In contrast, the expression of anti-inflammatory cytokines TGF-β and IL-10 were significantly elevated (Supplemental Fig. 4A, B). These findings meant BAFF-Trap could regulate inflammatory cytokine production in joints.

### BAFF-Trap suppresses activation of T cells, enhances the roles of regulatory T cells, and attenuates Th1 and Th17 cell functions

**Fig. 6:**
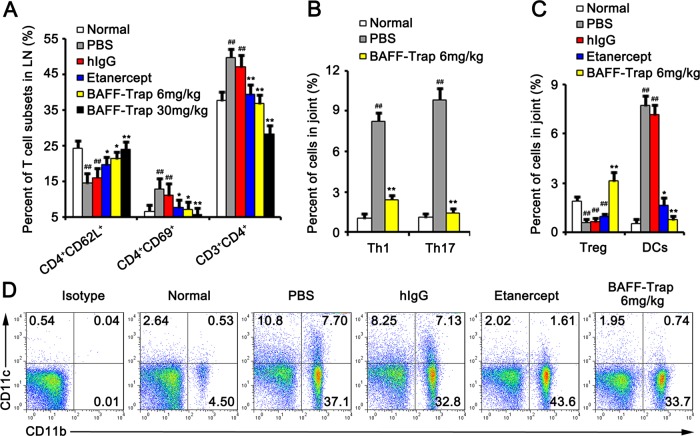
BAFF-Trap regulates the activation and polarization of T cells, suppresses the activation of DCs. **a** Percent of T-cell subsets in axillary and popliteal lymph nodes. Bars represent mean ± S.D. (*n* = 5). On day 42 after the indicated treatments, mice were killed to obtain lymph nodes. Then the cells in lymph nodes were incubated with corresponding antibodies to detect the subsets of T cells. Bars represent mean ± S.D. (*n* = 5). **b** Percentage of Th1 and Th17 cells in total joint cells. On day 42 after treatment, the joints were obtained and digested by collagenase type IV. Then Th1 (CD4^+^IFN-γ^+^) and Th17 (CD4^+^IL-17^+^) cells from joints were analysed by FACS. Bars represent mean ± S.D. (*n* = 5). **c** Percentage of Treg cells and DCs in total joint cells. The joint cells were obtained as mentioned in **b**. CD4^+^CD25^+^Foxp3^+^ meant Treg cells, CD11b^+^CD11c^+^ meant activated DCs. Bars represent mean ± S.D. (*n* = 5). **d** Representative FACS analysis of DCs. **P < 0.01 versus PBS; ^##^*P* < 0.01 versus normal

TACI and Br3 are expressed by B cells and activated T cells. Moreover, TACI–BAFF interactions are important in the regulation of T-cell activation in the presence of anti-CD3 mAb.^30^ Activated T cells are essential for CIA generation and development.^25^ To determine whether the effect of BAFF-Trap on CIA disease was related to the inhibition of T-cell activation, lymph nodes were collected from CIA mice and lymphocytes isolated. The number of CD4^+^ T cells was significantly decreased in mice treated with BAFF-Trap or Etanercept compared with PBS and hIgG treatment groups (Fig. 6a). Analysis of expression markers on CD4^+^ T cells demonstrated lower expression of activation markers (CD69) and higher expression of unactivated T-cell marker CD62L on CD4^+^ T cells from mice treated with BAFF-Trap compared with control mice (Fig. 6a). Thus, BAFF-Trap markedly suppressed T-cell activation, which is important in inhibiting CIA development.

Inflammatory cytokines can regulate T-cell polarization, and their production can be influenced by BAFF-Trap. To determine whether T-cell polarization was inhibited by BAFF-Trap, Th1, and Th17 cells in the joints were detected by FACS. In BAFF-Trap-treated mice, significantly reduced numbers of Th1 cells were observed in the joints (Fig. 6b). Semiquantitative RT-PCR assay revealed that mRNA levels of TNF-α, IFN-γ, IL-2, and IL-12 were downregulated. However, the expression of IL-10 was markedly elevated. Th1 differentiation is induced by IFN-γ and IL-12, whereas IL-10 suppresses cytokine production by Th1 cells and Th1 cell-mediated immune responses.^31^ IL-10-secreting cell (Treg and Breg cells) numbers in the joints were significantly elevated in BAFF-Trap-treated mice compared with controls (Figs. 4c and 6c).

IL-10 produced by regulatory lymphocytes can also suppress the production and differentiation of Th17 cells, which play an important role in the development of CIA disease.^28,31^ To determine the effect of BAFF-Trap on Th17 cells, we measured the number of Th17 cells in joints. As expected, the production of Th17 cells and IL-17 mRNA was significantly suppressed in BAFF-Trap-treated mice (Fig. 6b). Joints of BAFF-Trap-treated mice also showed significantly reduced levels of IL-6, TNF-α, and IL-1β, which contribute to the differentiation of Th17 cells. Interestingly, although an autocrine or paracrine source of TGF-β can promote generation of Th17 cells, the level of TGF-β in joints was markedly elevated. These data indicate that BAFF-Trap directly and indirectly suppresses the activation and differentiation of T cells.

### BAFF-Trap inhibits the generation of DCs in CIA

DCs are antigen-presenting cells that induce naive T-cell activation and contribute to the expansion of activated T cells.^32^ However, the number and function of DCs can be suppressed by regulatory lymphocytes secreting IL-10 or TGF-β.^28^ To determine whether DCs were influenced by BAFF-Trap through IL-10 and TGF-β, DCs in the joints were detected by FACS. The numbers of DCs in PBS and hIgG groups were higher than that in normal mice. However, BAFF-Trap markedly suppressed the production of DCs, which was dose-dependent (Fig. 6c, d).

### BAFF-Trap suppresses the BAFF-triggered NF-κB pathway

**Fig. 7:**
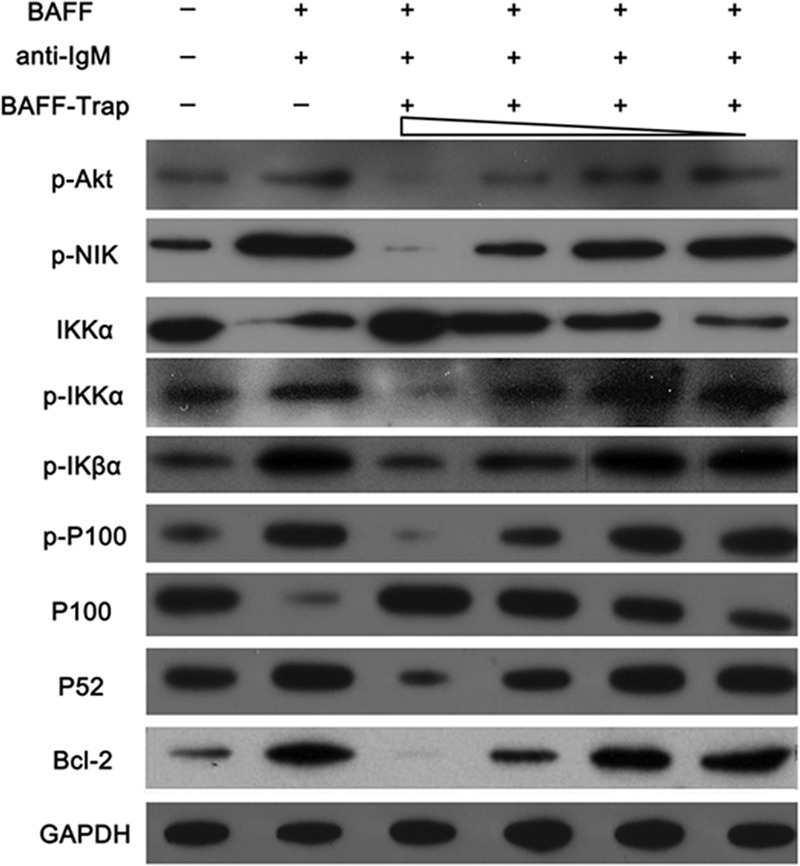
BAFF-Trap suppresses the activation of canonical and noncanonical NF-κB-signaling pathways triggered by BAFF and anti-IgM. Raji cells were cultured in the presence of BAFF (100 ng/ml) and anti-IgM (10 μg/ml), along with various concentrations of BAFF-Trap (1.56, 6.25, 25, and 100 μg/ml). After 48 h, the canonical and noncanonical NF-κB-signaling pathways were detected by western blotting

B-cell survival and maturation are mainly mediated by the BAFF/Br3 system, which induces activation of the canonical and noncanonical NF-κB-signaling pathways.^33,34^ Moreover, TACI can bind to TNFR-associated factor (TRAF) 2, 5, and 6, contributing to NF-κB activation. To determine whether BAFF-Trap suppressed NF-κB activation induced by BAFF/Br3 or BAFF/TACI systems, we treated Raji cells with BAFF, anti-IgM, plus serial concentrations of BAFF-Trap, and examined NF-κB activation by detecting the phosphorylation of IκBα and processing of p100. Consistent with previous reports, Raji cells showed a significantly strong response to the stimulation of BAFF and anti-IgM, as demonstrated by elevated phosphorylation of IκBα. When treated with BAFF-Trap, Raji cells showed markedly reduced levels of p-IκBα, which had a distinct dose–response curve (Fig. 7).

BAFF-Trap significantly inhibited the phosphorylation of p100 and the processing of p100 to p52 induced by BAFF and anti-IgM (Fig. 7). The phosphorylation of NIK and IKKα were also significantly suppressed by BAFF-Trap. Furthermore, in Raji cells treated with BAFF-Trap, the level of Bcl-2 was markedly reduced compared with control cells, which inhibited the antiapoptotic ability of autoreactive lymphocytes (Fig. 7). Thus, BAFF-Trap suppressed the activation of NF-κB-signaling pathways, and inhibited lymphocyte survival and maturation.

## Discussion

BAFF has an important role in the generation and development of RA and CIA. When BAFF binds its receptors it induces B-cell maturation, survival and development, but also contributes the activation of T cells, a hallmark of autoimmune diseases.^25,30,35^ Thus, BAFF is an ideal target for autoimmune diseases associated with lymphocyte hyperactivity, and the neutralization or elimination of BAFF can significantly ameliorate disease severity or suppress the development of autoimmune disorders.^36^

**Fig. 8:**
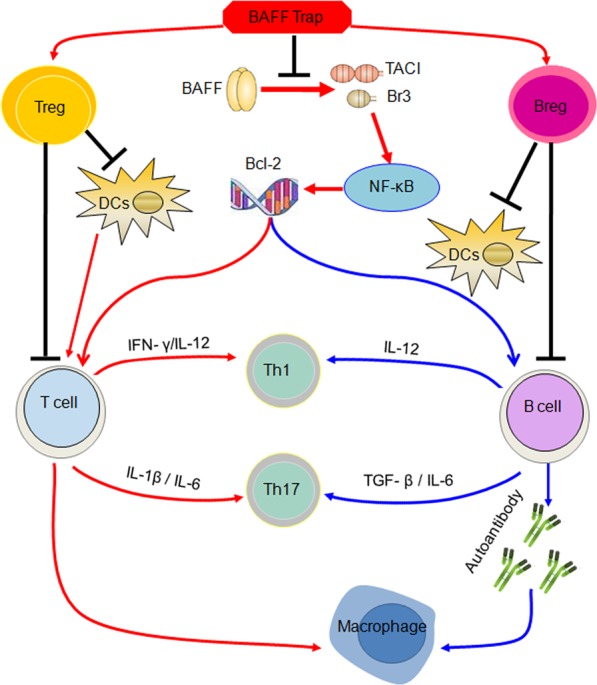
Simplified scheme proposing the function of BAFF-Trap in treating collagen-induced arthritis (CIA). BAFF-Trap inhibits BAFF from binding toTACI and Br3, and then suppresses the activation of NF-κB pathway. Activated NF-κB induces Bcl-2 production, which triggers the activation of T and B cells and promotes the development of B cells. Moreover, Bcl-2 contributes to the production of inflammatory cytokines from B cells, which regulate the polarization of T cells. Besides these, plasma cells producing autoantibodies and activated T cells contribute to the polarization of macrophages. Autoreactive B cells, activated T cells and M1 macrophages play an important role in the generation and development of CIA. Although the reasons that BAFF-Trap induces the production of Breg and Treg cells are not clarified, Breg and Treg cells can inhibit the activation of DCs, B and T cells, which contribute to the development of CIA

In this study, we described the development of a novel BAFF antagonist, BAFF-Trap, to treat CIA and AIA. BAFF-Trap binds to different species of BAFF, but not APRIL, whose role is still undefined in RA. Although the affinity of BAFF-Trap for BAFF was slightly lower than TACI-Fc, it was higher than that of Br3-Fc. The bioactivity of therapeutic proteins is key for treating diseases. Our data show that BAFF-Trap had markedly higher bioactivity than TACI-Fc and Br3-Fc for the suppression of Raji cell proliferation, indicating BAFF-Trap might be superior to other BAFF receptor antagonists. Furthermore, BAFF-Trap inhibited lymphocyte activation triggered by NF-κB-signaling pathways, and ameliorated synovial inflammation and joint destruction in CIA and AIA model (Figs. 7 and 8). Based on these findings, BAFF-Trap may be a novel and potential therapeutic protein for the treatment of RA.

In addition to BCR and CD40-mediated signals, B-lymphocyte survival and maturation requires activation of the BAFF/Br3 system pathway.^37^ After stimulation with BAFF, Br3 preferentially induces the noncanonical NF-κB pathway through binding to TRAF3, contributing to the processing of p100 to p52.^33,34^ Recent studies showed the noncanonical NF-κB-signaling pathway triggered by Br3 is NIK and IKKα-dependent, and inhibition of Br3 levels suppressed NIK activation and IKKα phosphorylation.^38^ Our results showed that BAFF significantly elevated the phosphorylation of NIK and IKKα proteins. However, neutralization of BAFF with BAFF-Trap decreased BAFF levels and suppressed activation of the noncanonical NF-κB-signaling pathway. Moreover, the activation of NIK and IKKα proteins was significantly inhibited, and p100 processing to p52 was almost completely blocked. BAFF also promotes B-cell survival through the canonical NF-κB-signaling pathway, which involves the activation of IKK complex and degradation of IκBα.^34^ Although IKKα is associated with NIK, which takes part in activation of the noncanonical NF-κB pathway as a component of classical IKK complex, its activation also induces the degradation of IκBα that releases p50.

BAFF-triggered NF-κB activation upregulates the expression of antiapoptotic factors, such as Bcl-2, Bcl-x_L_, and Bfl-1/A1, which contribute to B-cell survival and maturation.^33,38^ The process of B-cell development is regulated by the balance between B-cell proliferation and apoptosis. In RA patients and CIA mice, B-cell apoptosis is suppressed, contributing to the development of immature B cells and activation of mature B cells. Elevated levels of p52 and Br3 are found in T2 and mature B cells, whereas lower levels are observed in T1 cells.^38,39^ Moreover, the Btk/c-Rel pathway enhances the expression of Br3 and p100.^40^ This indicates that BAFF preferentially induces the survival of T2 and mature B cells. This is supported by evidence that loss of BAFF or Br3 results in significant downregulation of T2 and mature B cells, with no obvious changes in T1 B cells. However, significantly reduced numbers of immature transitional B cells (CD19^+^IgM^+^) were found in our study. This might be explained by CD19^+^IgM^+^ B cells being mainly T2 B cells in the spleens of mice treated with BAFF-Trap.

Besides producing immunoglobulins, mature B cells can present antigens to T cells and secrete cytokines.^28,31^ B cells can function as antigen-presenting cells and deliver co-stimulatory signals (e.g., CD40/CD40L) to T cells, which contributes to T-cell activation.^25,31^ Moreover, B cells secrete cytokines that regulate the function of T cells, which may occur in autoimmune conditions.^31^ There are two kinds of B cells: effector B cells and Bregs. Effector B cells secrete IFN-γ and IL-12 that promote Th1 differentiation.^28,31^ The production of IL-6 and TGF-β by B cells is important for Th17 cell differentiation.^11,41^ In addition, B cells can also indirectly regulate T-cell function through activating DCs. Interestingly, T-cell polarization amplifies the levels of these cytokines, contributing to the production and development of CIA disease.^31^ Nevertheless, Breg cells can suppress T cell responses, but enhance the generation of Tregs, mainly via IL-10 and TGF-β. Breg cells can also inhibit the activation of DCs and macrophages through IL-10.^28^ Recent studies also showed that TACI and Br3 are not only expressed on the surface of B cells but also on the surface of T cells. Soluble BAFF binds to T cells contributing to T cell activation.^25,30^ Thus, our findings show that upregulation of BAFF in mice substantially increases the secretion of proinflammatory cytokines and the proportion of Th1 and Th17 cells, which was ameliorated through administration of BAFF-Trap (Fig. 8).

Although several strategies targeting BAFF have been used to inhibit autoreactive B-cell activation and ameliorate autoimmune disease, the heterogeneity of patient responses and side effects have limited the development and application of these strategies. Although belimumab was therapeutic in a clinical phase I/II study in RA patients and was approved by the FDA to treat SLE patients, patient heterogeneity has limited its clinical phase III study.^13^ Meanwhile, Atacicept (TACI-Fc) can cause infections, related to the rapid decline of serum IgG.^13,42^ In this study, we did not observe obvious immunopathology in mice treated with BAFF-Trap. The levels of Breg and Treg cells were significantly elevated, although some studies have described that approaches targeting B cells can also reduce Breg cells. However, the level of BAFF was elevated in all CIA mice, which was normalized after treatment with BAFF-Trap. However, not all patients are responsive to BAFF-dependent B-cell-directed therapy, and the role of BAFF-Trap in CIA mice should be further studied ^13,42^.

In conclusion, this study demonstrated that BAFF-Trap effectively reduced the levels of autoimmune IgG and activated lymphocytes, and suppressed the development of CIA and AIA without serious side effects. Thus, BAFF-Trap may be a novel and effective strategy to treat autoimmune disorders.

## Supplementary information

Supplemental Table and Figure
