# Supplementary material for: Therapeutic effects of a novel BAFF blocker on arthritis
Source: Signal Transduct Target Ther. 2019 Jun 14;4:19. doi: 10.1038/s41392-019-0051-z (PMC6565627; doi:10.1038/s41392-019-0051-z)
Supplement: Supplementary file 1 — Supplemental Table and Figure [file 41392_2019_51_MOESM1_ESM.doc]

**Supplemental Table 1**. Primers for semiquantitative RT-PCR

| **Target gene** | **Forward / Reverse** | **Primer sequence (5'-3')** | **Size (dp)** | **Annealing temperature (˚C)** |
| --- | --- | --- | --- | --- |
| IFN-γ | Forward | CACTGCATCTTGGCTTTGC | 411 | 66 |
|  | Reverse | AACAGCTGGTGGACCACTC |  |  |
| TNF-α | Forward | TGGAACTGGCAGAAGAGGC | 463 | 68 |
|  | Reverse | GAGATAGCAAATCGGCTGAC |  |  |
| BAFF | Forward | TGTTGTCCAGCAGTTTCAC | 347 | 62 |
|  | Reverse | CTGCAGACAGTCTTGAATGA |  |  |
| β-Actin | Forward | GCATTGCTGACAGGATGC | 166 | 68 |
|  | Reverse | CCTGCTTGCTGATCCACATC |  |  |
| IL-1β | Forward | TGGCAACTGTTCCTGAACTC | 320 | 64 |
|  | Reverse | CCATGAGTCACAGAGGAT |  |  |
| IL-6 | Forward | TCACAGAGGATACCACTC | 336 | 68 |
|  | Reverse | CTGTATCTCTCTGAAGGAC |  |  |
| IL-2 | Forward | AGCTCGCATCCTGTGTCAC | 469 | 68 |
|  | Reverse | TGACAGAAGGCTATCCATC |  |  |
| IL-17 | Forward | GAGAGCTTCATC TGTGTCTC | 331 | 58 |
|  | Reverse | ATTCATGTGGTGGTCCAGC |  |  |
| IL-12 | Forward | TTGT AGAGGTGGACTGGAC | 326 | 68 |
|  | Reverse | TGAGCACGTGAACCGTCC |  |  |
| APRIL | Forward | actgatccaacagacagagc | 419 | 60 |
|  | Reverse | cttgtccttcccgagatac |  |  |
| TGF-β | Forward | ACGGAAGCGCATCGAAGCCATC | 557 | 68 |
|  | Reverse | TGCTGTCACAAGAGCAGTGAGC |  |  |
| IL-10 | Forward | GCTGGACAACATACTGCTAAC | 322 | 68 |
|  | Reverse | GCAGTTGATGAAGATGTC |  |  |

IFN-γ = interferon gamma; TNF-α = tumor necrosis factor alpha; BAFF = B-cell activating factor; IL-1β = interleukin-1 beta; IL-6 = interleukin 6; IL-2 = interleukin 2; IL-17 = interleukin 17; IL-12 = interleukin 12; APRIL = a proliferation-inducing ligand; TGF-β = transforming growth factor beta; IL-10 = interleukin 10


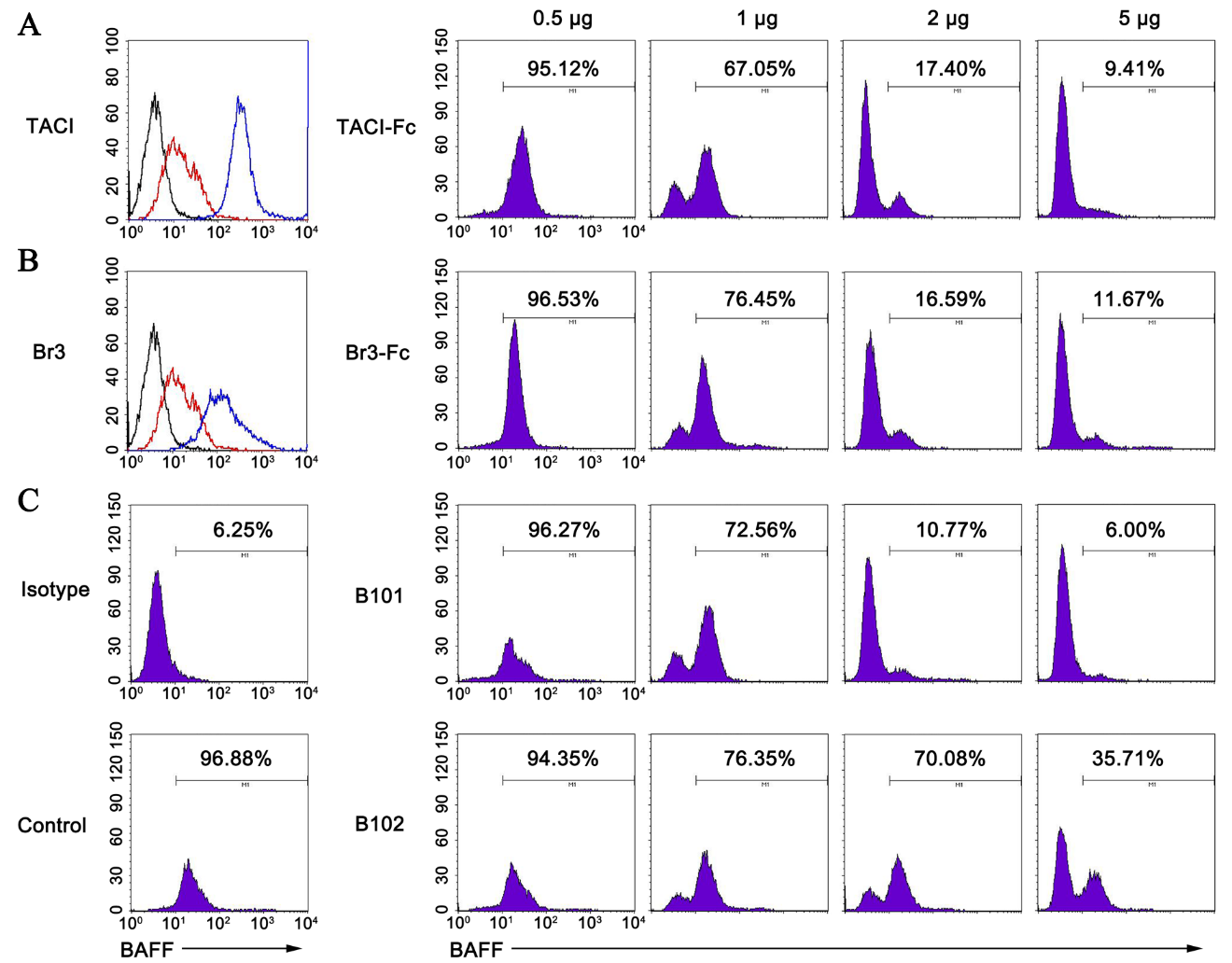


**Supplemental Fig. 1. BAFF-Trap inhibits BAFF to bind Raji cells expressing TACI and Br3.** (A) The expression of TACI on the surface of Raji cells. (B) The expression of Br3 on the surface of Raji cells. (C) BAFF-Trap inhibits BAFF binding to Raji cells.


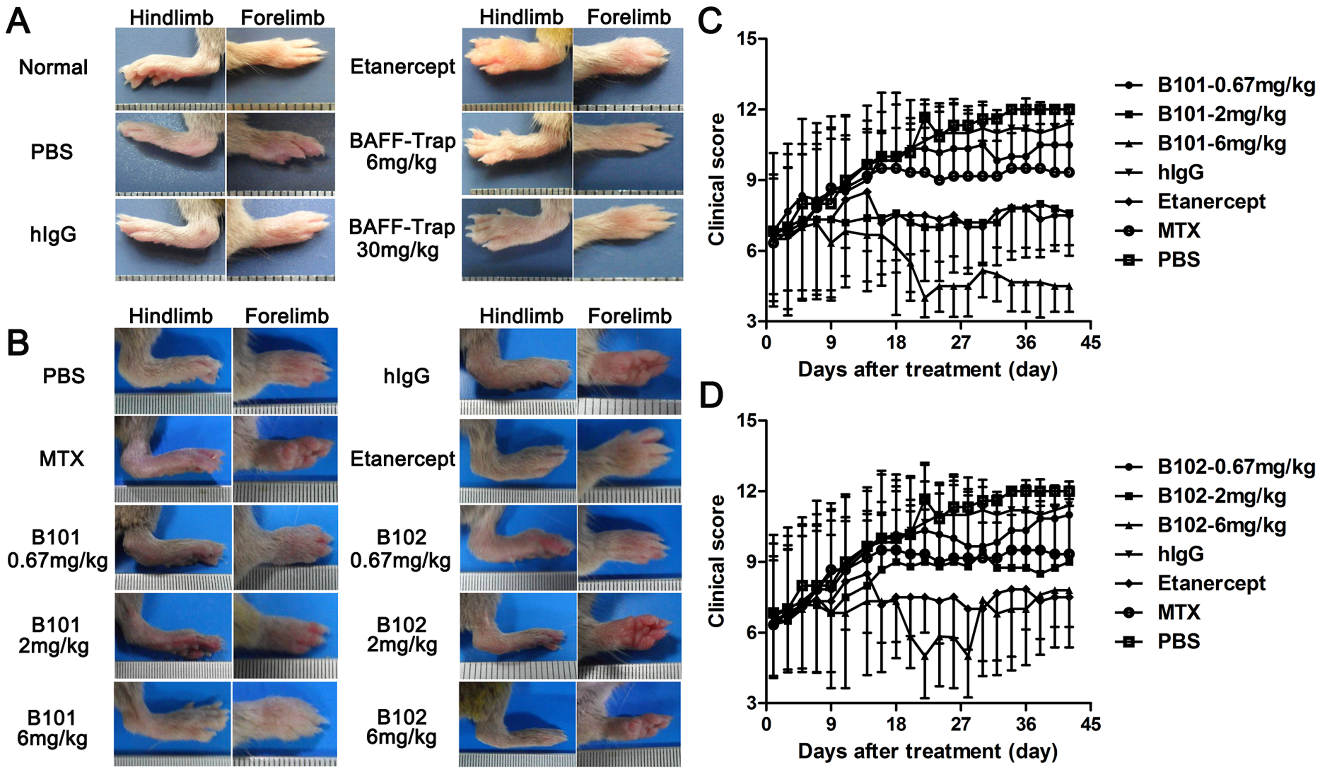


**Supplemental Fig. 2. Detection of joint appearance and clinical score in CIA mice treated with BAFF-Trap**. (A-B) Detection of joint appearance in CIA mice (n=5). (A) On day 42 after treatment with 6mg/kg or 30mg/kg of BAFF-Trap (B101), mice were narcotized and the joints were photoed. (B) On day 42 after treatment with 0.67mg/kg, 2mg/kg or 6mg/kg of B101 or B102, mice were narcotized and the joints were photoed. **(**C-D**)** Clinical score of CIA mice treated with serial concentrations of B101 and B102 (0.67 mg/kg, 2 mg/kg, and 6 mg/kg) (n=5). (C) The clinical score of mice treated with B101, and (D) represents that treated with B102. 6 mg/kg and 2 mg/kg of B101 significantly suppress the development of CIA, while the clinical score in mice treated with B102 was only decreased in 6 mg/kg of B102 treatments.


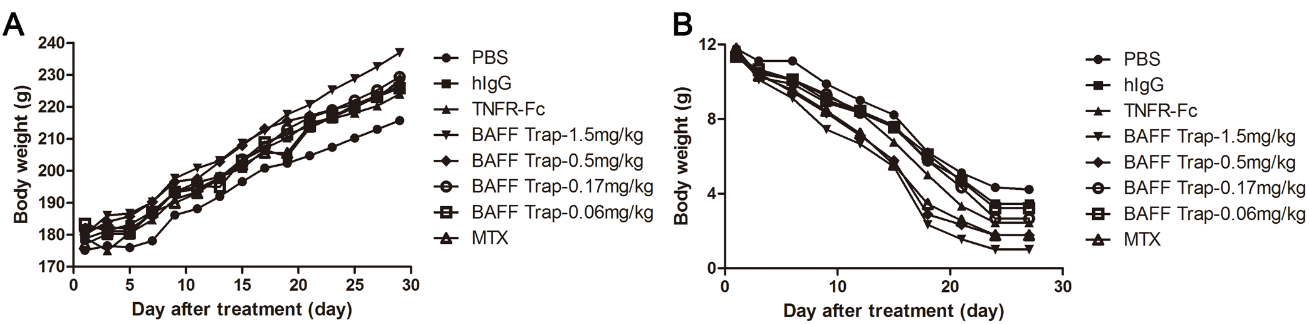


**Supplemental Fig. 3. Detection of effective dose of BAFF Trap in AIA model.** Rats were treated with high clinical score (n=9)**.** (A) Body weight of AIA rats. (B) Clinical score of AIA rats.


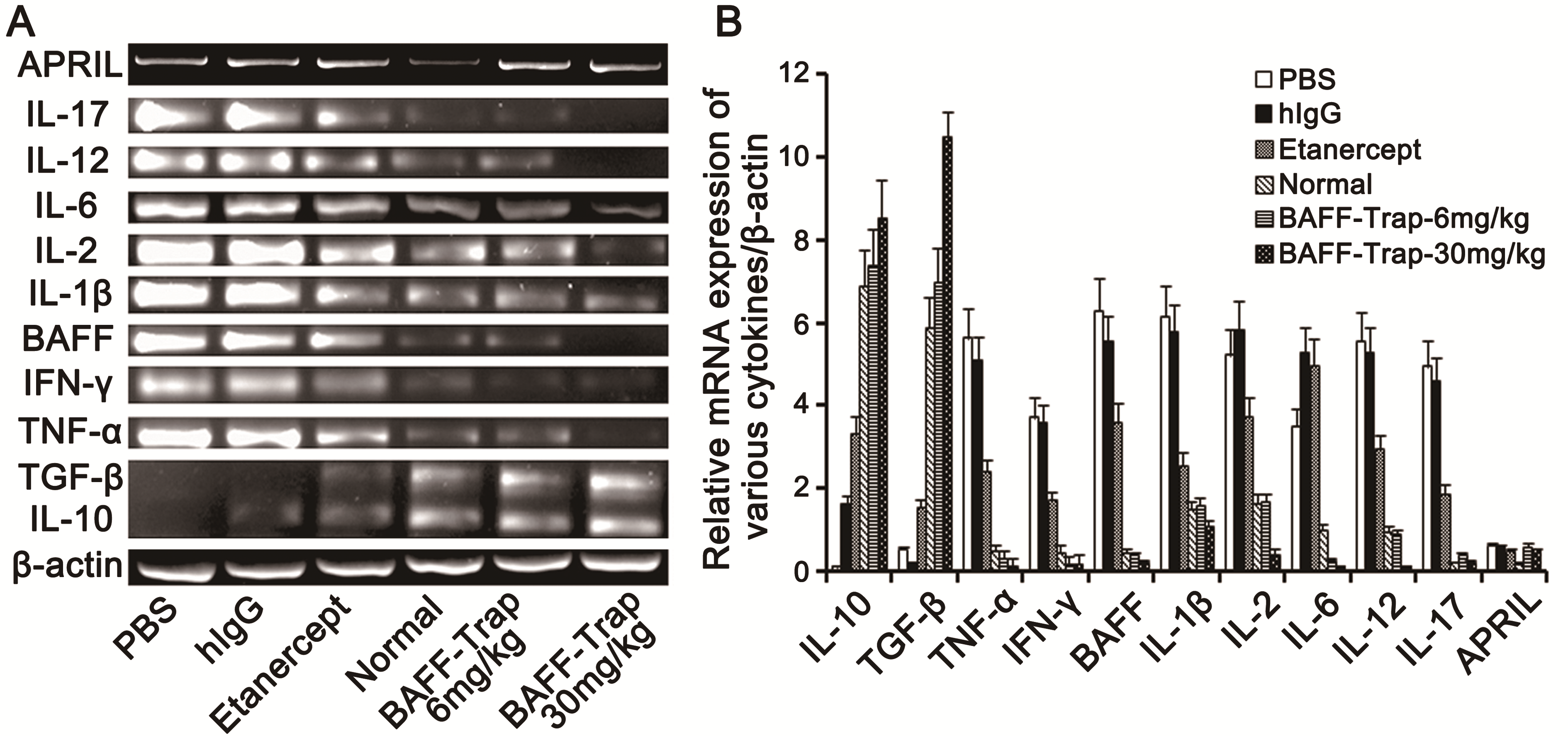


**Supplementary Fig. 4. Detection of gene levels of cytokines from joints in CIA mice.** (A) Gene levels of cytokines from joints. On day 42 after treatment with BAFF-Trap, total RNA was extracted from representative joints of CIA mice with Trizol, and then the gene leves of inflammatory related cytokines were detected by reverse transcription and semiquantitative PCR. (B) Relative ratios of these cytokines to β-actin are determined by densitometric analysis.
